# Repeated Listening Increases the Liking for Music Regardless of Its Complexity: Implications for the Appreciation and Aesthetics of Music

**DOI:** 10.3389/fnins.2017.00147

**Published:** 2017-03-31

**Authors:** Guy Madison, Gunilla Schiölde

**Affiliations:** ^1^Department of Psychology, Umeå UniversityUmeå, Sweden; ^2^Västmanland Hospital in VästeråsVästerås, Sweden

**Keywords:** appreciation, aesthetics, complexity, familiarity, liking, mere exposure, music, preference

## Abstract

Psychological and aesthetic theories predict that music is appreciated at optimal, peak levels of familiarity and complexity, and that appreciation of music exhibits an inverted U-shaped relationship with familiarity as well as complexity. Because increased familiarity conceivably leads to improved processing and less perceived complexity, we test whether there is an interaction between familiarity and complexity. Specifically, increased familiarity should render the music subjectively less complex, and therefore move the apex of the U curve toward greater complexity. A naturalistic listening experiment was conducted, featuring 40 music examples (ME) divided by experts into 4 levels of complexity prior to the main experiment. The MEs were presented 28 times each across a period of approximately 4 weeks, and individual ratings were assessed throughout the experiment. Ratings of liking increased monotonically with repeated listening at all levels of complexity; both the simplest and the most complex MEs were liked more as a function of listening time, without any indication of a U-shaped relation. Although the MEs were previously unknown to the participants, the strongest predictor of liking was familiarity in terms of having listened to similar music before, i.e., familiarity with musical style. We conclude that familiarity is the single most important variable for explaining differences in liking among music, regardless of the complexity of the music.

## Introduction

Music preferences and their underpinnings have a long history in psychology (e.g., Meyer, 1903; Washburn et al., 1927; Krugman, 1943; Seashore, 1947; Cattell and Saunders, 1954). Historically, the grounds for such preferences have largely been sought in structural aspects of the music, in characteristics of listeners, and in interactions between these domains. Aesthetic theory argues that novelty, surprise, and continuous development is central to the appreciation of works of art, including music (Meyer, 1956, 2001; Berlyne, 1971). For more recent discussions, see Kivy (1990) and Scruton (1997). According to Berlyne (1971), the hedonic value of music is related to optimal levels of arousal. Specifically, the music listener is rewarded or feel pleasure as a result of reduced arousal through the relief of an unpleasant curiosity or, alternatively, of moderate increments in arousal by exploration. Both cases involve a relief or relaxation of uncertainty following an act of exploration induced by curiosity.

Following the insights of Darwin (1871), the foundation of musical phenomena is also sought in our evolution, and their mapping upon possible adaptive functions in the present or in the past (e.g., Madison, 2011, 2014; Miller, 2011; Charlton et al., 2012; Huron, 2012; Ravignani et al., 2014; Merker et al., 2015; Mosing et al., 2015; Savage et al., 2015).

In neurological terms, pleasurable musical experiences seem to be related to the dopaminergic reward system, important loci of which include the amygdala, midbrain, ventral striatum, orbitofrontal cortex, and ventral medial prefrontal cortex (e.g., Blood and Zatorre, 2001). Unfamiliar music that elicited pleasant feelings was associated with activation in the anterior insula, cingulate gyrus, hippocampus, nucleus accumbens, and prefrontal anterior cingulate (Brown et al., 2004). Music experiences are also reflected in neuroendocrine changes. For example, listening to techno-music—but not classical music—increased the heart rate, systolic blood pressure, and concentrations of several neurotransmitters, peptides, and hormones related to emotional states (Gerra et al., 1998). Both fulfillment and violation of expectations can be seen as central aspects of learning, which is proposed to account for their connection to pleasure in a computational model based on dopaminergic neurons and predictive coding (Gebauer et al., 2012; cf. Menon and Levitin, 2005). Consistent with this, musical processing is faster and more accurate when it is harmonically related to preceding stimuli, related to FMRI activation in the inferior frontal gyrus, frontal operculum, and insula (Tillmann et al., 2003). Repeated listening to a 3-min piece of music increased the overall EEG power across these 3 min, although the change across the repetitions was not reported. Subjective ratings of valence and arousal decreased across repetitions, however (Jäncke et al., 2015). Processing of music-syntactic features as such has been reported in Broca's area and its homolog in the right hemisphere (Maess et al., 2001).

An obvious tenet of the optimal arousal model is that too simple or too familiar music would tend to be perceived as trivial and boring, while too complex or unfamiliar music would tend to be incomprehensible. Both would theoretically lead to a loss of interest and liking. This implies that people would grow tired of the same old works and desire unheard music constantly, but this is not what we see. On the contrary do people in general like the music they are already familiar with most of all, as is well known by the world's hard-working cover artists. In fact, a majority of the world's professional musicians earn their living by playing tunes over and over again that are already frequently heard in concerts and on the radio, and found on recordings in millions of homes.

Today, familiarity with the music is acknowledged as a central factor for liking, which is seen in listening history as well as genre exposure (e.g., Krugman, 1943; Edmonston, 1969; Heingartner and Hall, 1974; Peery and Peery, 1986; Hargreaves, 1988; Fung, 1996; North and Hargreaves, 1997). For example, popular music has become less complex and more homogeneous in terms of more restricted pitch transitions and less varied timber since the 1960s (Serra et al., 2012). If there is an inverse association between complexity and familiarity, less complex music will attract more listeners initially. They will, on the other hand, sooner lose interest as their familiarity with it increases, because it was initially closer to the threshold of boredom. This may however be in the interest of the music industry, because both a greater turnover rate and a larger body of initial listeners will lead to greater revenue, provided it can supply still new music at a relatively low cost. Conversely, more complex music will require more listening to gain the level of familiarity required to like it, and only a small minority of potential listeners will either appreciate it from the start or be inclined to listen to it until they begin to appreciate it. Thus, one can see how this secular change might reflect economic considerations in the light of technical developments that allow greater production, public exposure, and sales to a smaller cost per unit. These observations show how patterns of relationships between complexity and liking may be of both practical and societal relevance.

Folk psychology also contains the notion that music can be more or less complex, based on individual differences in liking and motivation to engage in such music, and that such preferences are related to differences in training, personality, or intellectual ability (see e.g., Gaver and Mandler, 1987). It makes intuitive sense that one will tend to dislike music that is beyond one's level of comprehension, or more formally, processing ability. Likewise, it seems feasible that music which has a complexity level substantially below one's processing ability might be experienced as tedious, repetitive, and boring, and will thus be disliked. These two aspects are captured in Berlyne (1971) inverted U curve, also known as the Wundt curve (Wundt, 1874).

**Figure 1:**
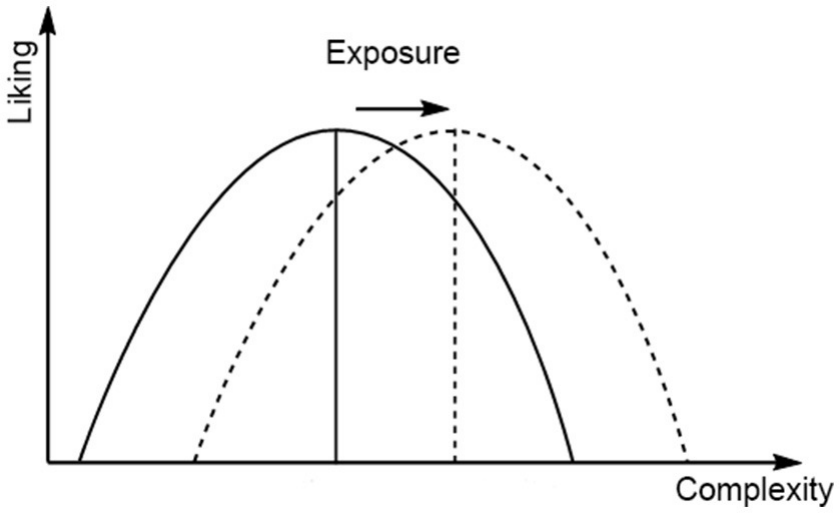
**The Wundt curve, and its hypothesized movement to the right with increased exposure (i.e., repeated listening)**.

Combining complexity with familiarity, the simple idea addressed in the present study is that liking of more complex music should increase with more listening, whereas liking for less complex music should decrease with more listening. Specifically, on the basis that more listening implies more training, which increases the individual's processing ability, we hypothesize that repeated listening should move the apex of the U curve for liking as a function of complexity to the right, that is, toward greater complexity, as illustrated in Figure 1.

There are several more or less formalized accounts of the cognitive mechanisms underlying liking as a function of repeated listening, with or without the interaction with complexity (for reviews see Berlyne, 1971; Bornstein, 1989). The “mere exposure” effect is central to most of these (for reviews see Zajonc, 1968, 2001; Harrison, 1977). However, the central concepts of these approaches are not formulated in such a concrete level of detail that they can be precisely applied to music or musical properties, and results have also been inconsistent (Zajonc et al., 1974; Martindale et al., 1988; Bornstein, 1989; Brentar et al., 1994). This suggests that a formalization in terms of these theoretical frameworks would be both difficult to achieve and limited in its reach. Rather, we conclude that there is a robust empirical increase in liking with familiarity, and that it is inevitably, at least to some extent, related to increased processing ability (e.g., Krugman, 1943; Bradley, 1971; Heingartner and Hall, 1974; Hicks and King, 2011).

Critical issues for the present study are to choose the appropriate numbers of presentations, to determine levels of complexity, and to maintain a high degree of ecological validity. These factors are therefore discussed in some detail below, reviewing previous research that has been considered in arriving at the two-step design of the present study.

### Design considerations

Previous empirical studies which have used repeated music examples (MEs) to assess familiarity, complexity, and liking have produced varied results. In some cases only one repetition has been found to increase the liking for real as well as synthetic MEs (Bradley, 1971; Peretz et al., 1998). Others have found that liking only increased after a specific number of presentations (Mull, 1940, 1957; Bartlett, 1973; Heingartner and Hall, 1974). In contrast, some studies have also found a decrease in liking after several presentations (Coppock, 1978; Brentar et al., 1994; Szpunar et al., 2004; Schellenberg et al., 2007), and still in other cases multiple presentations yielded no change in liking at all (Obermiller, 1985). In general, it seems that the most common result of repeated listening is increased liking, whereas decreased liking occurs mainly when MEs are monophonic, synthetic, or repeated within the same listening session.

Musical complexity is notoriously difficult to define and formalize. Complexity can refer to both aspects of the structure as well as the listener's subjective interpretation based on their experience. While there are a number of clear and precise definitions of complexity, such as entropy or Shannon's measure of information (Shannon and Weaver, 1949), their application to music is problematic. It is not understood how the perception of a piece of music is construed by the brain, and how it could be modeled by a formal mathematical measure of complexity. Several studies have nevertheless attempted objective evaluations of complexity, by using information-theory based indices to synthesize varyingly complex MEs (Vitz, 1966; Heyduk, 1975) or attributing perceived complexity to quantifiable properties *ad-hoc* (Conley, 1981; Hargreaves, 1984; Rohner, 1985). One way to apply formal measures of complexity would be an experimental situation in which complexity could be independently and exclusively manipulated. This level of control, however, requires highly constrained sound sequences, and synthetic examples typically have low ecological validity (Heyduk, 1975; Steck and Machotka, 1975; Arkes et al., 1986; Hargreaves and Castell, 1987; Szpunar et al., 2004). Another approach would be to separate listeners into groups based on their cognitive processing abilities while keeping the musical complexity constant, but results from such studies have not indicated any preference among those with more advanced abstract thinking for more complex music during either focused listening or listening during a cognitive demanding task (Rohner, 1985; Arkes et al., 1986).

On the other hand, music al complexity has considerable face validity if one considers, for example, short children's songs with few pitches and note values in contrast to avant-garde music with many harmonically distant pitches and rhythmical syncopes. A more ecologically valid approach to determine musical complexity may therefore be to let humans subjectively rate it. This approach has been used in several previous studies (cf. Burke and Gridley, 1990; North and Hargreaves, 1995). Some studies have relied on the listeners to rate complexity before (Burke and Gridley, 1990; Brentar et al., 1994) or after (North and Hargreaves, 1995; Orr and Ohlsson, 2005) the experiment, or by asking musicians to produce improvizations with different levels of complexity (Orr and Ohlsson, 2001). However, when complexity is not experimentally manipulated but rated in the same session and by the same listeners who also rated their liking, effects may well be a result of confounds between complexity, familiarity, and liking (Orr and Ohlsson, 2001).

Considering the problems of mapping structural properties to complexity reviewed above, and that this approach also requires synthetic MEs with poor ecological validity, we concluded that careful pre-experimental selection according to perceived complexity was the best option.

Regarding the musical structure of the MEs, possible effects of autobiographical associations from previous listening were avoided by selecting MEs that listeners had never heard before their participation, as these were not part of our hypotheses. Another important concern was to emulate the conditions in which people normally listen to music. The experiment was, for practical reasons, limited to approximately 4 weeks in order to decrease the risk for attrition and fatigue. We gauged that hearing each ME once per day was reasonable, in view of the relatively large number of MEs required to vary complexity in four levels and still have a sufficiently large number of MEs within each level to diffuse their idiosyncratic properties. Adults safely beyond adolescence were selected as participants in order to have acquired a modicum of experience of different musical styles and to some extent have overcome the focus on using music preferences as a means for identification with a group or with certain values, which some adolescents exhibit (Finnäs, 1993; Sloboda, 2001). The music was selected to be at least superficially familiar to people in this age-segment, similar to popular music likely to be heard on the radio, and mainly featuring the standard electrified instrumentation characteristic for pop and rock music.

Finally, we concluded from previous research that pre-experimental selection of music examples and the determination of their complexity should be done by another group of participants than those who rate familiarity and liking, to avoid misattribution of correlations among complexity, familiarity, and liking (Orr and Ohlsson, 2001).

In conclusion, the present study assesses the liking of music examples as a function of their complexity and exposure. Complexity and familiarity were factorially varied such as to yield a large number of potentially optimal combinations, and thus maximize the opportunity to observe an interaction between complexity and repeated listening.

### Hypotheses

The main hypotheses were that (1) liking exhibits a Wundt curve relationship with the number of presentations, (2) liking exhibits a Wundt curve relationship with complexity, and (3) repeated listening should move the apex of the Wundt curve for liking as a function of complexity toward greater complexity. Previous results indicate that another likely outcome is (4) that liking increases monotonically with the number of presentations without any interaction with complexity, in accord with preferences for people and many other stimuli (Sluckin et al., 1982; Martindale et al., 1988; Reber et al., 2004).

## Selection of music examples

Because the selection of music examples and determination of distinct levels of complexity is critical for the rating experiment, we will describe these procedures in some detail.

### Materials and methods

#### Participants

Eight musicians were recruited to rate the level of complexity of music as a basis for selecting examples to be used in the repeated listening experiment. Musical experts were expected to appreciate different structural elements in the complex stimulus stream (Brown et al., 2015) and have a much broader experience of different musical styles than lay listeners, who were expected to base their ratings on style preferences to a greater extent (Coggiola, 2004). The experts were four men and four women between 28 and 47 years old (*M* = 34.3, *SD* = 4.9). All were musically educated (6–12 years) and currently performing musicians, as well as professionally engaged as musicians, music teachers, composers, or arrangers (Orr and Ohlsson, 2005). Preferences for higher levels of complexity are not correlated with greater musical experience (Arkes et al., 1986), so no potential confounds are introduced by the use of experts for initial complexity rating.

#### Initial music selection

As mentioned in the introduction, prime requirements for the repeated listening experiment were that the MEs should be familiar in style and surface properties, while the particular MEs should be unknown to the participants. This precludes sampling of MEs from representative fora, such as radio, hit lists, and so forth. Moreover, since complexity is a study variable, MEs should cover a wide range of musical complexity. We defined a set of inclusion and exclusion criteria and applied these to our own collections of audiograms, comprising approximately 800 albums. Inclusion criteria were (1) music which was generally characteristic of popular music in terms of musical properties as well as instrumentation and (2) foreign elements for a Western audience were accepted only if combined with more familiar elements in the accompaniment. Exclusion criteria were (3) styles distinctly different from pop-, rock-, jazz-, and world music or from any mix of these styles, and (4) traditional folk music unless featuring said accompaniment. In addition, (5) music with vocals was excluded in order to avoid that vocal quality and lyrics would become confounding variables (Fung, 1996; Coggiola, 2004). Finally, (6) MEs assumed to have been frequently played in broadcast media or to otherwise be widely known were also excluded to preclude as far as possible that the experts had previously heard them. This also decreased the risk that social conventions would affect ratings (Fisher, 1951; Crozier, 1997).

From each of the tracks selected in this fashion, one or in some cases two excerpts of 25–100 s in duration were copied. Each of those 197 MEs were meant to constitute an independent musical statement, comprising for example a complete passage or phrase. MEs were taken from an instrumental part of the track in case vocals were included in the track, often the introduction or the bridge. The instrumentation consisted mainly of electric or acoustic guitar, bass and percussion. Many examples also featured piano or different electric keyboard instruments as well as melodic instruments like saxophone, trombone, or violin.

Author GS selected 80 out of these 197 MEs so as to maximize the range of complexity across all styles (Listed in Supplementary Material, Appendix A), and the selected MEs were then edited in duration. Longer MEs were shortened and shorter MEs were lengthened by repeating sections so that all MEs ended up between 38 and 75 s in duration. Abrupt beginnings or endings were softened with fade-ins or fade-outs, and all MEs were adjusted to an equal loudness level. Finally, the 80 MEs were recorded on CDs in four different random orders.

#### Rating procedure

The experts received two CDs containing the 80 music examples, written instructions, and a paper form on which to indicate familiar MEs. They were instructed to listen to all the music examples once in the order they appeared on the CDs and to indicate on the form if they recognized any example or if they could identify any composer or performing artist.

Within 2 days of completed listening of the CDs, each expert was summoned to an individual session and was instructed to rate each ME on eight dimensions. A custom application run on a laptop computer presented them as eight visual analog scales on the screen in the form of horizontal lines with a slider. Each scale was anchored “Do not agree at all” and “Fully agree,” in response to the following statements: *I like this music example, The music is of high quality, The overall impression is that the music is complex, The melody is complex, The harmony is complex, The music contains complex rhythms, The tempo is high*, and *The instrumentation is extensive/unusual*. This type of ratings is frequently used in the music psychology literature (e.g., Gabrielsson, 1973; Hargreaves et al., 1980; Obermiller, 1985; Balkwill and Thompson, 1999; Davies et al., 2013; Frühauf et al., 2013; Kawakami and Furukawa, 2013; Madison and Sioros, 2014; Sioros et al., 2014; Witek et al., 2014).

The scales appeared in a different random order for each ME, and MEs were presented in an individual random order for each expert. Experts were asked to indicate on a paper form if they found any ME to deviate from the others in terms of style, sound quality or any other property. The task was completed in each individual's own pace, with the opportunity to take breaks as desired, and lasted between 2 and 3 h discounting breaks. Finally the expert's musical education, music experience, and music preferences were recorded.

### Results and discussion

The distribution of the ratings were approximately normal for all scales, with no skewness (range −0.35 to 0.09), but trending toward being platycurtic (range −0.97 to −0.34). Because visual analog scales produce data that correspond to an interval scale (e.g., Lukacs et al., 2004), one-way mixed ANOVAs were applied to assess the effects of ME. From lowest to highest, F_79, 553_ was 2.96 for Instrumentation, 3.08 for Liking, 5.85 for Overall complexity, 5.94 for Quality, 6.05 for Harmonic complexity, 6.17 for Melodic complexity, 11.55 for Rhythmic complexity, and 18.68 for Tempo (all *p* < 0.00001). Inter-rater reliability was assessed with Cronbach's alpha, which was 0.661 for Instrumentation, 0.676 for Liking, 0.831 for Quality, 0.834 for Overall complexity, 0.835 for Harmonic complexity, 0.838 for Melodic complexity, 0.913 for Rhythmic complexity, and 0.946 for Tempo. We also assessed whether the mean of Melodic, Harmonic, and Rhythmic complexity would exhibit higher reliability (0.841) than the Overall complexity rating (0.834), but the difference was marginal.

Finally, we explored which weights experts assigned to the four complexity components in rating the overall complexity by means of multiple linear regression. Simultaneous regression models indicated that Melodic complexity was the strongest predictor of Overall complexity, followed by Harmonic and Rhythmic complexity and Instrumentation, while Quality and to some extent Liking also appeared to make significant contributions, depending on the particular set of predictors included. Considering that explicit complexity components should theoretically be more important than Quality and Liking, a stepwise forward regression was performed on the 80 mean ratings across experts. The final model included 6 of the 7 predictors and accounted for 95.3% of the variance (multiple *r* = 0.976). Melodic complexity accounted for 86.5% (ß = 0.401) of the variance, followed by Rhythmic complexity (ß = 0.201, *r*^2^ change = 6.4%), quality (ß = 0.362, *r*^2^ change = 1.3%), Instrumentation (ß = 0.100, *r*^2^ change = 0.5%), and Tempo (ß = 0.105, *r*^2^ change = 0.2%), while Harmonic complexity was not entered in the model.

These results indicate that melodic and rhythmic complexity are independent predictors, while harmonic complexity was presumably subsumed by melodic complexity for these short MEs. The high model *r*^2^ suggests that experts were consistent in their attribution of complexity, although inter-rater reliability was not that high. A conclusive validation of the ratings can only be made in relation to another group of participants, however, which will be done in connection with the main experiment.

#### Final selection procedure

All 80 MEs were rank ordered according to the overall complexity and to the mean of the rhythmic and melodic complexity, both of which led to almost the same order. Considering also an even distribution of musical styles, 10 MEs close to the 13th, 37th, 62nd, and 87th percentile were selected. The mean complexity rating z-scores for each of these four groups were −1.0, −0.4, 0.2, and 1.1. The difference between adjacent levels of complexity varied thus from 0.6 to 0.9 standard deviations according to the expert ratings.

## Main experiment

The purpose of this experiment was to address the interaction between the number of presentations and the levels of complexity determined in Experiment 1 with regard to liking. Independent variables were *Complexity* in four levels, number of *Presentations* in four levels (1, 10, 19, and 28), and *ME* in 10 levels, and the main dependent variable was *Liking*. Three additional rating scales were included for the main purpose of validating the experiment. First, we wanted to validate the complexity rated by the experts in the selection study by means of ratings by the musically less experienced participants, without disclosing that this was a variable of interest. We chose two Swedish words related to complexity, namely “konstigt” and “enformigt.” The first can be translated as odd, strange, or intricate and the second as dull, repetitive, or monotonous; in what follows they will be called *Odd* and *Dull*. Second, it is important to assess the level of *Familiarity*, since that has been found to be strongly related to liking (e.g., Berlyne, 1970; Harrison, 1977; Bornstein, 1989; Zajonc, 2001). In the first rating session participants were asked to rate how familiar the MEs were, and on remaining sessions to rate if they had recently listened to similar music apart from the MEs.

### Materials and methods

#### Participants

Inclusion criteria were an interest for music and willingness to devote the time and effort required for the present study, which made it possible to use a convenience sample. The participants were recruited amongst music professionals acquainted with author GS, some of whom added additional participants from their own acquaintances. The final group consisted of 10 women and five men between 28 and 70 years of age (*M* = 50.7, *SD* = 11.1), with a wide range of musical experience and music listening habits. All reported normal hearing, and had between 2 and 10 years tertiary education. Four participants listened mainly to pop, rock, ballads, and other popular music, five listened more often to jazz-, folk-, world-, vocal, and classical art music, while the remaining six listened to even wider ranges of styles. Four participants listened mainly to music in the background while doing other chores (incidental listening); five practiced mainly focused listening, while the remaining participants practiced both. Ten participants normally listened to music between half an hour and 10 h a day, while the rest listened less often than that. Six participants had none or only sporadic formal musical training, while the others had taken lessons in music theory or musical performance, or had attended music classes in school sometime between the ages of 10 and 18. This heterogeneity was considered appropriate, since any consistent results across participants could be considered more generalizable than those obtained for a more homogenous group.

#### Rating scales and materials

Using the same equipment, interactive rating application, and general set-up as for the experts, the participants rated the following statements on visual analog scales: *I like this music example, This music example is odd* (“konstigt”), *This music example is dull* (“enformigt”). The first session also featured the statement *I listen to similar music*, which in subsequent sessions was replaced by *I have recently listened to similar music*, explained as referring to music listened to outside of the study. The 40 music examples were recorded onto CDs in seven different randomized orders marked with the weekdays Monday through Sunday (Listed in Supplementary Material, Appendix B). Participants used their own sound reproduction systems for listening at their preferred locations; at home, in the car etc.

#### Procedure

The participants were informed both orally and in writing that they should listen to all MEs on the CD marked with the corresponding day once every day of the week, except on the day scheduled for a rating session. No restrictions were placed on when and where this listening took place. The first time that the participant heard each MEs was through the laptop computer on the first rating session, when they also had to indicate on a paper form if they recognized any ME or artist. Rating sessions took place in the participant's home with the experimenter present, using the same software as in Experiment 1, which played the MEs in a randomized order. After that the participants listened to the MEs on their own once a day for 8 days, and then again participated in a rating session. If for any reason listening did not occur on a given day, participants were instructed to listen to that CD on the following day, in addition to the CD scheduled for that day. This procedure was repeated so that four ratings were obtained, with eight individual listening sessions with no ratings in between. For the participants who followed the timetable, the study lasted for 4 weeks with a total of 28 presentations. Participants were also told to record each listening occasion at the time it took place in a diary provided by the experimenter. Participants were not informed about the purpose or the hypotheses of the study, or that the music examples had been selected for different levels of complexity. Care was taken not to mention or in any way suggest that complexity was an issue, in order to avoid possible demand characteristic bias.

#### Statistical analyses

Mixed ANOVAs were used to analyse the effects, and linear multiple regression was used to assess the proportion of variance accounted for by each independent variable. The ratings were considered as interval scale data, because they were obtained with visual analog scales (e.g., Lukacs et al., 2004). The critical outcome of the dependent variables was their trend across the four rating sessions, not contrasts between any two sessions. Adjusting for multiple comparisons was therefore not relevant.

### Results

The diaries showed that the participants for the most part followed the daily listening schedule. Five participants delayed the schedule for various reasons from 1 to 3 days. One participant stopped listening due to illness, but resumed after 26 days. Two participants listened one time less than requested. The participants mainly listened alone in everyday situations where they simultaneously performed household chores, ate, bathed, cleaned, read, used a computer, rode in or drove a car, or worked. The cases when the participants listened together with others were few and mainly involved family situations, having friends over or when in a car with others. One participant engaged in concerted listening without any concurrent activity.

**Figure 2:**
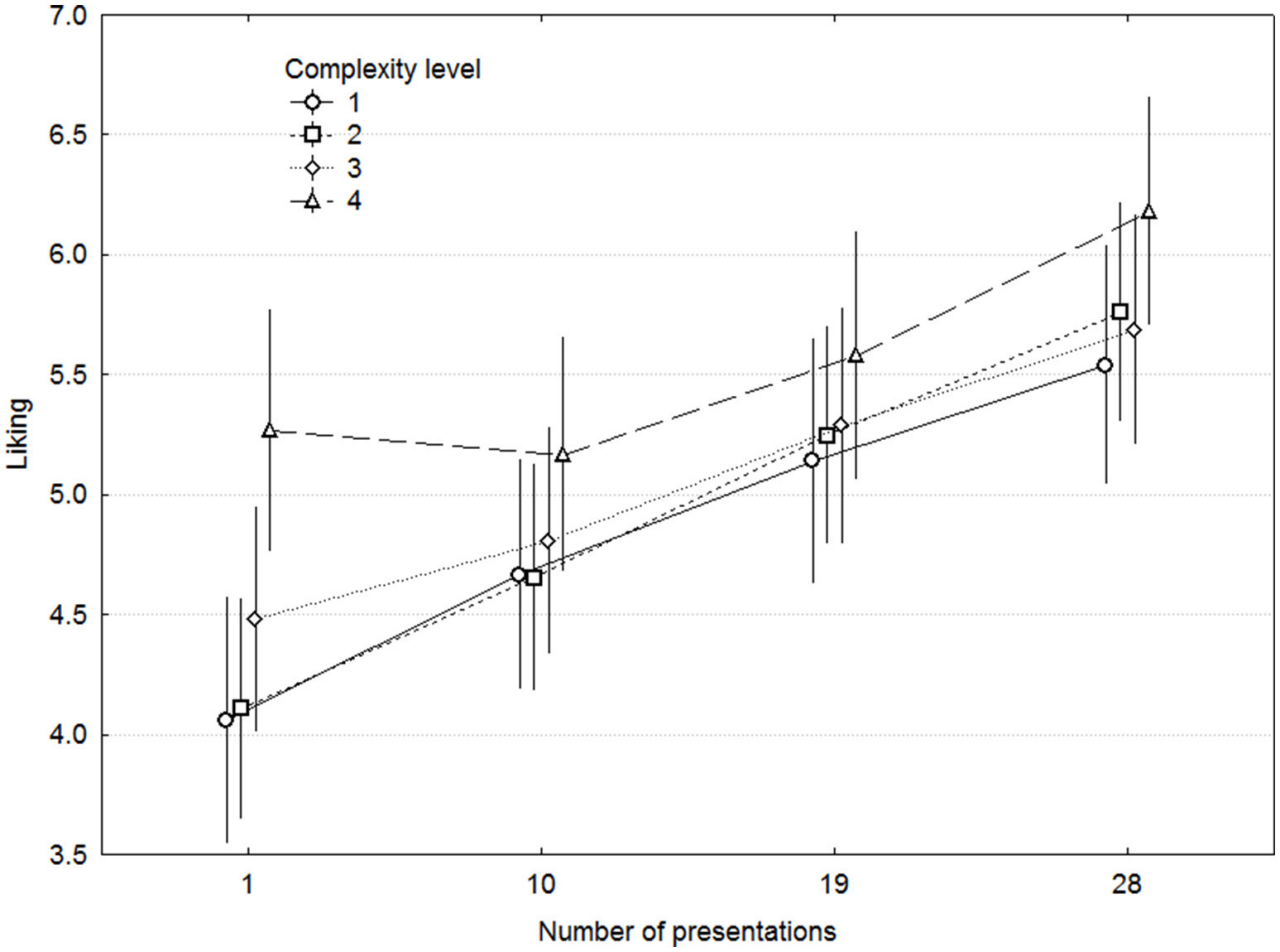
**Ratings of liking as a function of complexity and presentations**. Error bars denote 0.95% confidence intervals.

The grand mean of all ratings was close to 5, indicating that participants used the scales in a symmetric fashion. Grand means for each scale indicated that MEs overall were found to be moderate in Liking, low to moderate in Dull and Familiar, and low in Odd. The distribution of the ratings was approximately normal for all scales. For the last session, Skewness and Kurtosis were −0.38 and −0.85 for Liking, 1.24 and −0.71 for Odd, 0.25 and −1.21 for Dull, and 1.49 and 1.00 for Familiar. Liking increased with the number of Presentations but was not affected by Complexity, as seen in Figure 2. A three-way repeated-measures ANOVA demonstrated significant main effects of Presentations [*F*_(3, 42)_ = 12.35, *p* < 0.00001, ω^2^ = 0.197] but not of Complexity [*F*_(3, 42)_ = 0.81, *p* = 0.494], Presentations × Complexity [*F*_(9, 126)_ = 1.33, *p* = 0.225], nor any other interaction. ME was also included in the ANOVA, although it represents different ME in each level of complexity, in order to minimize error variance in the model [*F*_(9, 126)_ = 7.51, *p* < 0.00001].

We plotted the Presentations x Complexity interaction for each participant, which demonstrated a weak tendency for a decline in Liking at 19 or 28 Presentations for 4 out of 15 participants, and this was almost exclusively found for the two lowest Complexity levels. The largest decline was on the order of one scale unit, and was in no case statistically significant according to 0.95 confidence intervals.

Inter-scale correlations were 0.61 for Familiar × Liking, −0.38 for Familiar × Dull, −0.45 for Familiar × Odd, −0.39 for Liking × Odd, and −0.60 for Liking × Dull. All these correlations were computed on raw data from the first listening session (*N* = 600, all *p* < 0.0005).

**Figure 3:**
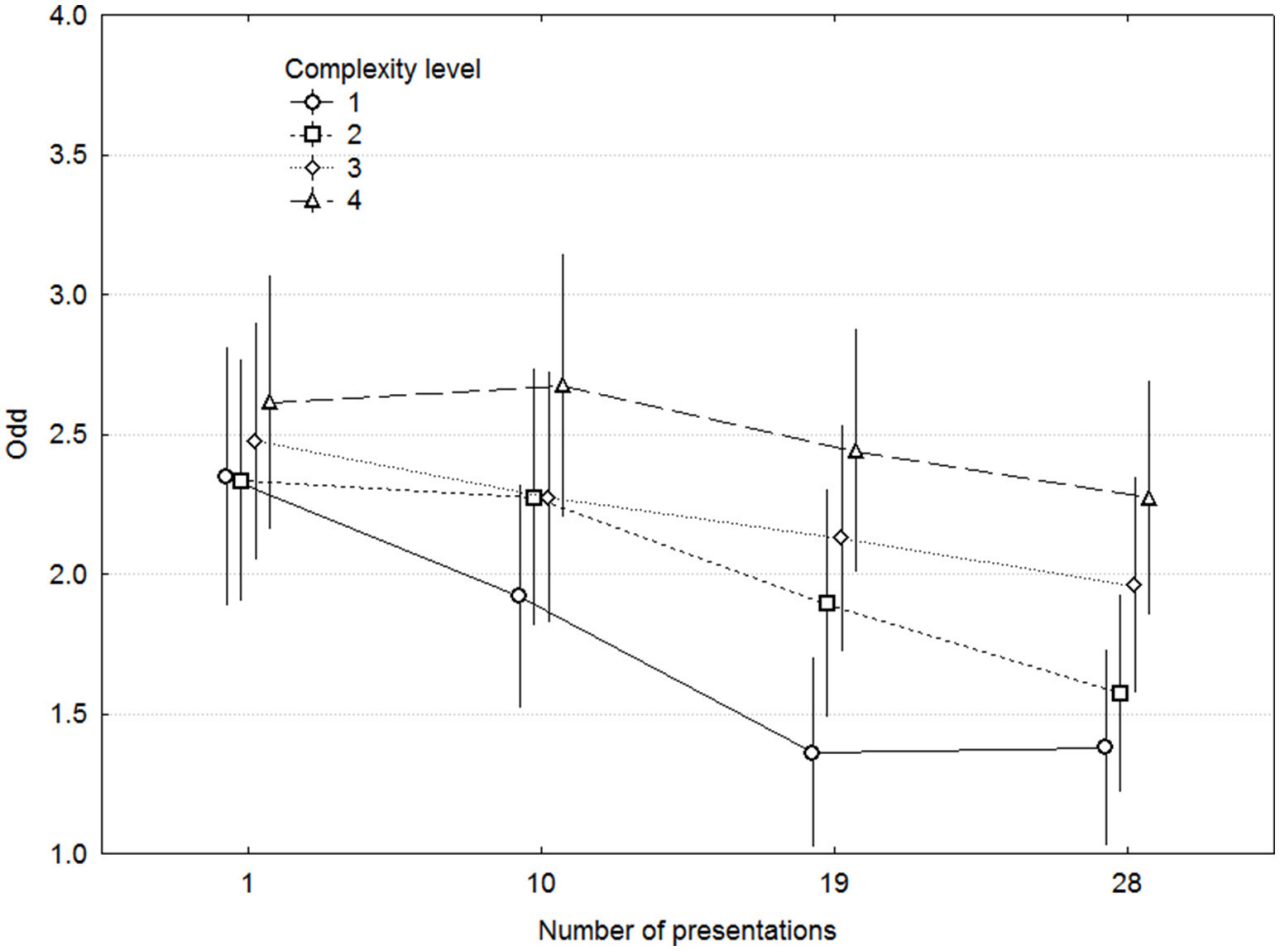
**Ratings of odd as a function of complexity and presentations**. Error bars denote 0.95% confidence intervals.

**Figure 4:**
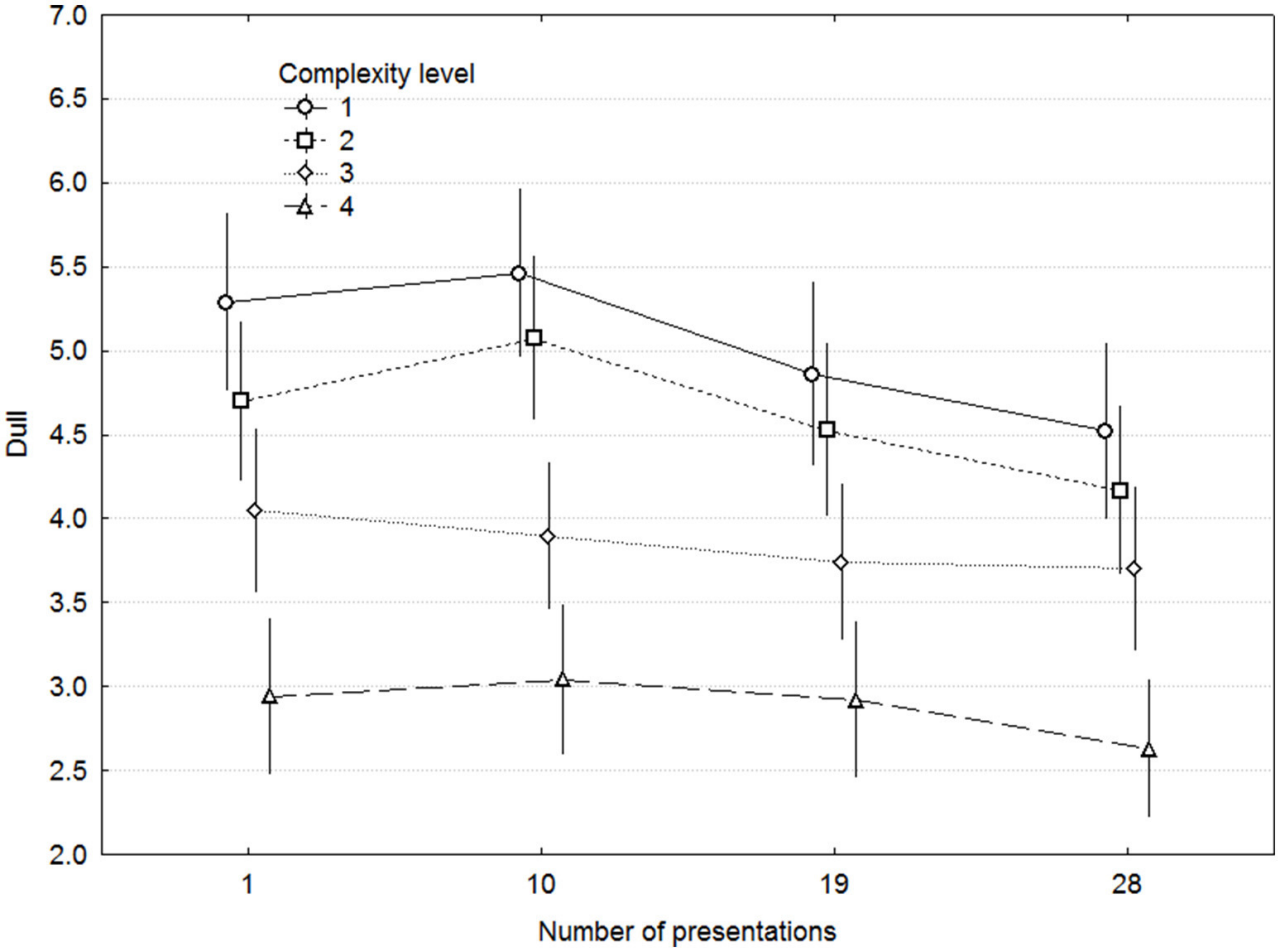
**Ratings of dull as a function of complexity and presentations**. Error bars denote 0.95% confidence intervals.

Mean ratings for Odd and Dull are shown in Figures 3, 4, and the considerably larger effects of Complexity on Dull than on Odd indicate that Dull captured the concept of complexity to a greater extent. The same kind of ANOVA as for Liking but with Dull as dependent variable demonstrated significant main effects of Complexity [*F*_(3, 42)_ = 11.09, *p* < 0.00005, ω^2^ = 0.346] and ME [*F*_(9, 126)_ = 10.95, *p* < 0.00001], but not of Presentations [*F*_(3, 42)_ = 2.365, *p* = 0.08] nor any other interaction except Complexity x ME, which was of no interest. For comparison, Odd exhibited largely the same pattern of effects, but the effect of Complexity was considerably weaker [*F*_(3, 42)_ = 3.27, *p* = 0.030, ω^2^ = 0.111]. More importantly, both Odd and Dull clearly reflect the levels of Complexity, with the least complex level being rated as least Odd and most Dull, and this pattern is consistent throughout all levels of Presentations. Simple correlations between the overall complexity ratings in Experiment 1 and the Odd and Dull ratings in Experiment 2 were 0.41 and −0.64 across the 40 MEs, and their correlations with other expert complexity ratings were in the same range (0.48–0.81).

To assess the validity of the ratings in a more systematic fashion, linear multiple regression was performed on the 40 means for each ME across participants in both studies, with the participants' Odd and Dull ratings as dependent variables and the experts' complexity components as predictors. Odd yielded over-fitted models that could not be interpreted, even when the number of predictors was reduced. Dull provided a good fit, however, in that a simultaneous model including all components explained 83.3% of the variance and indicated significant contributions of Liking and Harmonic complexity (both on the part of the experts). A stepwise forward regression model included only Melodic complexity out of the 5 predictors, and accounted for 49.3% of the variance (multiple *r* = 0.702).

**Figure 5:**
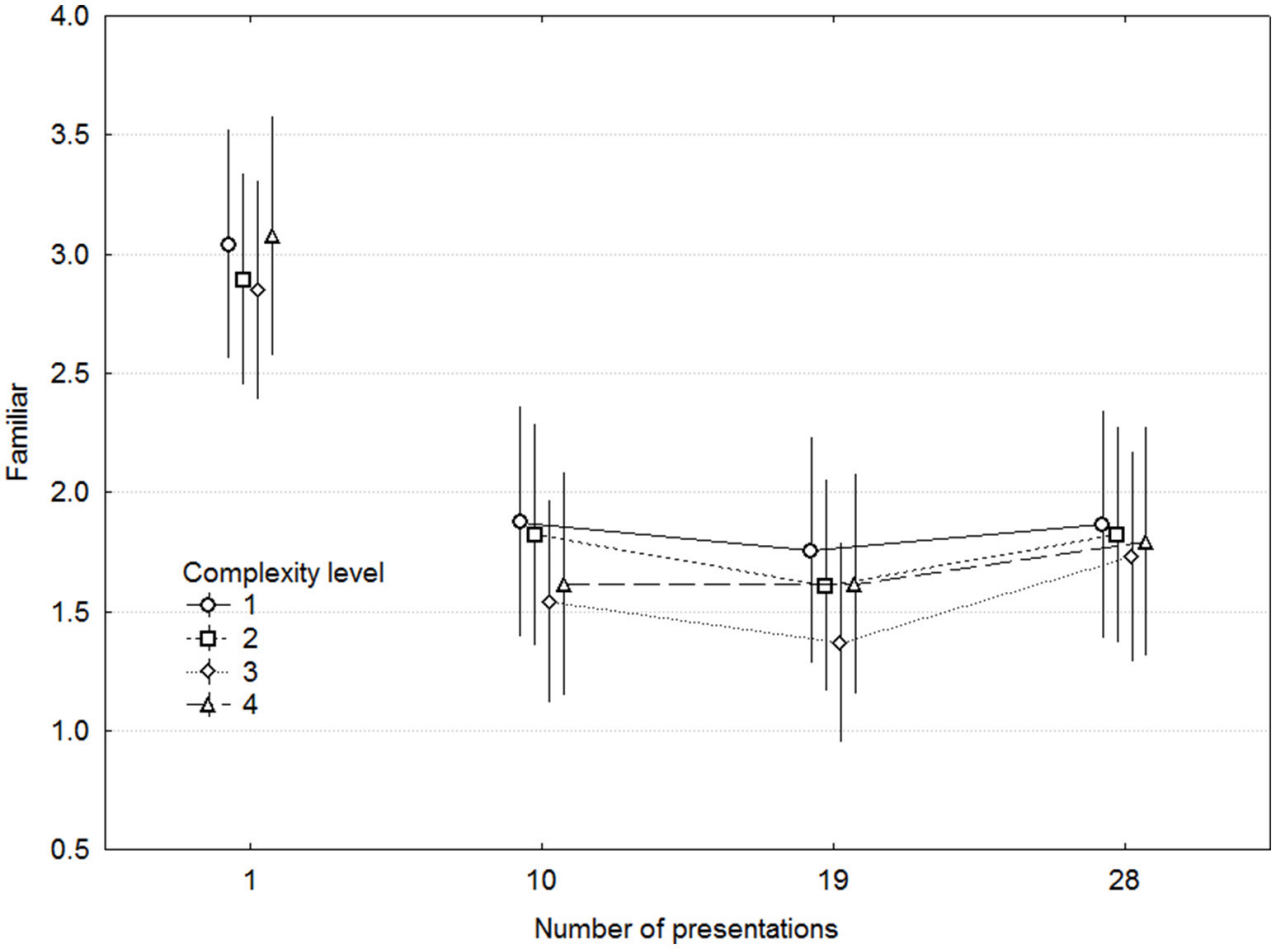
**Ratings of familiar as a function of complexity and presentations**. Error bars denote 0.95% confidence intervals. Note that different statements were rated in the first and in the subsequent rating sessions.

**Figure 6:**
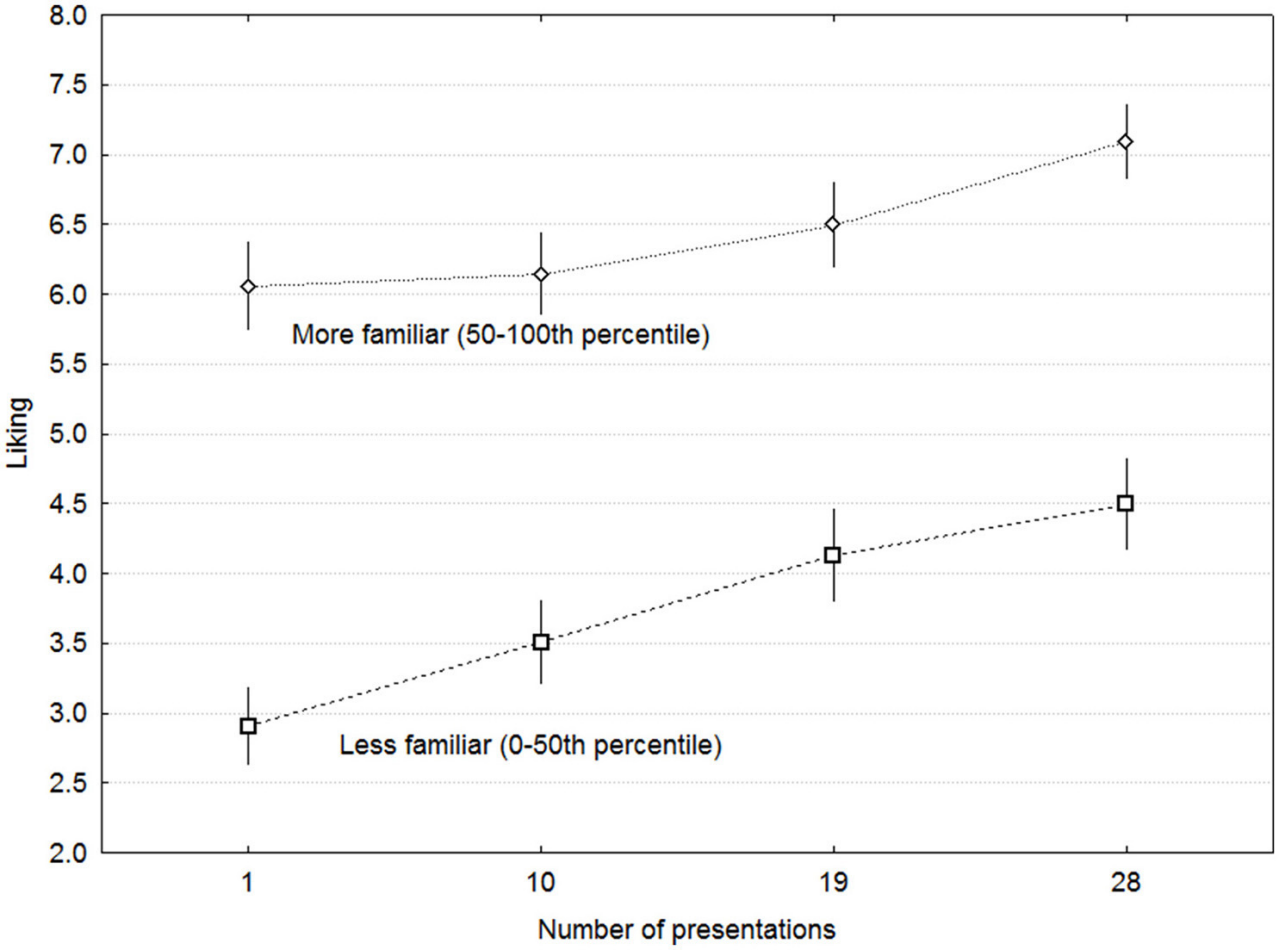
**Ratings of liking as a function of presentations and rated familiarity on the first presentation**. Error bars denote 0.95% confidence intervals.

Figure 5 shows the ratings of Familiarity as a function of Complexity and Presentations. Excluding the first rating session, which featured another statement than the subsequent ones, Familiarity was affected neither by Presentations [*F*_(2, 28)_ = 0.44, *p* = 0.64] nor by Complexity [*F*_(3, 42)_ = 0.150, *p* = 0.93] or any other interaction. Although there were no linear effects of any of these variables, there were nevertheless large differences in familiarity among MEs within each participant. To assess the effect of these, MEs were divided into two groups for each participant by the median of familiarity ratings on the first session. Liking as a function of Presentations is plotted separately for these groups in Figure 6, which demonstrates that the largest effect on Liking is attributable to initial familiarity in terms of having listened to similar music before.

These results indicate substantial linear contribution from both pre-experimental familiarity and within-experimental familiarity in terms of the number of presentations. It would therefore be informative to assess the relative amount of variance accounted for by complexity and the two forms of familiarity. A multiple linear regression model with mean ratings of Liking (range 0–10) across participants as the dependent variable yielded significant contributions of complexity levels (range of predictor variable 1–4, ß = 0.226), Presentations (range 1–28, ß = 0.464), and ratings of familiarity on the first session (range 0–10, ß = 0.551) as predictors (all *p* < 0.00005, *df* = 3, 156). The model *Liking* = 1.013 *Presentations* + 0.050 *Complexity* + 0.091 *Familiarity* accounted for 57.2% of the variance across MEs (multiple *r* = 0.756).

## General discussion

We examined the claim, consistent with popular belief, that appreciation for music peaks at intermediate levels of familiarity and complexity. The present study was designed to maximize the opportunity for a Wundt curve to manifest itself, both as a function of complexity, number of presentations, and of their interaction. The range of complexity was maximized within a sample of music that could still be characterized as popular, in terms of style and other surface properties. Differences between levels of complexity were further amplified by selecting MEs with distinct magnitudes of rated complexity. The number of presentations covered a range beyond that which has led to a decrease in liking in previous studies. Yet was no such effect found, but rather a monotonic increase in liking for both higher complexity and number of presentations.

Before discussing the results in detail, we will consider aspects of the study that might affect or limit its reach and conclusions. First, the initial audiograms came from the personal collections of the authors, and do not represent a random sample of all published music in a certain time period, for example. One can speculate that this circumstance rendered the MEs more likeable in the long-term course of listening employed. Also, the selection based on the experts' ratings favored MEs with low inter-rater variability, which might have imposed a bias on the remaining MEs. For example, complexity may be confused with prototypicality, inasmuch as stimuli that are typical for their kind are perceived as simpler regardless of their objective complexity (Martindale et al., 1988). As less familiarity with the style precludes the assessment of typicality this would lead to lower ratings of complexity. Yet, nothing in the results indicated that participants in the main experiment were more familiar than the experts with the jazz and progressive rock styles that dominated the highest complexity levels. The validity of the Complexity variable was supported by the correspondence between experts' ratings of complexity and listeners' ratings of Odd and Dull, even though the different terms would have invoked different concepts across the experiments. However, we cannot exclude the possibility that an inverted U for complexity might occur if an even wider range of complexity had been applied.

Second, the participants did not constitute a population sample. They had a relatively high involvement with music, which was probably a precondition for agreeing to listen to music for 40 min per day. Their higher interest for music might have disposed them to embrace even the unfamiliar examples to a greater degree. On the other hand, higher interest can also be associated with more well-defined preferences against certain styles or elements. Such preferences are recognized as having a strong effect (Hargreaves, 1984), but the present study suggests, on the contrary, that examples less liked initially were those that received the largest increase in liking with the number of presentations.

Third, we should consider the reliability and validity of the rating scales. It was not practically feasible to measure this with the present participants, because the experiment proper was so time-consuming in itself. But we also relied on the fact that a huge literature has used similar and in many cases almost identical scales and procedures for rating (e.g., Gabrielsson, 1973; Hargreaves et al., 1980; Obermiller, 1985; Balkwill and Thompson, 1999; Davies et al., 2013; Frühauf et al., 2013; Kawakami and Furukawa, 2013; Madison and Sioros, 2014; Sioros et al., 2014; Witek et al., 2014). In general, these studies indicate very high reliability of the ratings scales by cross-scale correlations up to 0.91 for trained participants (Hargreaves et al., 1980) and explained variance up to 0.90 in multiple regression models (Balkwill and Thompson, 1999) and factor analysis (e.g., Gabrielsson, 1973). Likewise, a substantial proportion (57%) of the variance was accounted for even across the fairly different dimensions rated in the present study.

That there was no decrease in liking with the number of presentations is not surprising, considering that each study that has shown this deviated in one or more ways from natural listening conditions. First, the same short ME was often heard several times in the same session (Mull, 1940, 1957; Hargreaves, 1984; Brentar et al., 1994; Jäncke et al., 2015). In the present study, the music and the frequency of listening were predetermined, but each ME was played only once in each session and the participants could choose the place, the time of day, and their concurrent activities. Second, the Western art music used in many previous studies is likely to yield very low liking ratings due to its minimal exposure in society for several decades, and in particular to younger people in student populations (Fisher, 1951; Bartlett, 1973; Coppock, 1978; Conley, 1981; Hargreaves, 1984; Peery and Peery, 1986; Burke and Gridley, 1990). In the present study, real music was selected to concur with musical styles familiar to the participants. There has been difficulty in interpreting the results of such studies as Orr and Ohlsson (2001), Steck and Machotka (1975) and Vitz (1966) who used unfamiliar improvizations and synthetic tone sequences corresponding to predetermined rules of complexity. These examples were consistently rated much lower than real music examples by participants, indicating low validity. Third, listening has sometimes been combined with concurrent laboratory tasks. Szpunar et al. (2004) presented speech in one ear and music in the other, and found monotonic increases in liking for up to 32 presentations of so-called incidental listening in the background, while liking decreased after 32 presentations during focused listening (during the same session). In comparison to the present results, one can clearly see a gradient of less natural listening, involving monophonic, synthetic, or short MEs, repeated within the same listening session, for example, that also corresponds with decreased liking.

In the present study, the listening conditions were allowed to vary among the 420 times (28 presentations × 15 participants) that each ME was heard, and the music may therefore have filled a different function at different times. The diaries and interviews indicate that participants largely listened to the stimuli as they would normally listen to music of their own choosing. Participants were often engaged in other concurrent activities, and one explanation for why liking of the least complex MEs remained high might be that this constituted an appropriate level of cognitive processing under those conditions (Konecni and Sargent-Pollock, 1976). The situation in which they actually made their ratings was considerably less natural, however. We cannot know if the ratings were affected by their listening in a more focused way during the rating task, but it is hard to find a methodological solution that avoids this. In conclusion, we feel that the ecological validity of the present study was higher than in previous studies, and more representative for typical music listening.

Nevertheless, the hypothesis that liking of more complex music benefits from more listening remains valid, and should manifest itself in a greater increase for more complex than for less complex music. This also failed to materialize. If anything, effects were greater for the less complex music, in terms of decreases in both Odd and Dull, while the increase in liking was just as large for all levels of complexity. One could argue that the absolute level of complexity was too low or the range of complexity was too small to confer such a dissociation. Neither of these explanations is consistent with the pattern of results, however. First, the main rating proxy for complexity—Dull—showed moderate but significant differences in the expected direction between each level of complexity. Second, too little complexity would have led to decreased liking, but this was not the case even for the more familiar MEs, as shown in Figure 6.

If we accept the present results, it should be noted that the most complex music was liked the most across all levels of presentations. If anything, a trend for the next most complex music to be liked next most suggests a U function rather than an inverted U. Support for this so-called optimal complexity hypothesis is reported by most studies that have explicitly tested it, either by directly manipulating one or both of these variables (Vitz, 1966; Heyduk, 1975; Hargreaves, 1984; Brentar et al., 1994; Orr and Ohlsson, 2001), or by inferring such a relationship indirectly (Bradley, 1971; Coppock, 1978; Sluckin et al., 1982; Obermiller, 1985; Burke and Gridley, 1990; North and Hargreaves, 1995; Peretz et al., 1998). Again, however, each of these studies differ in important design features that may explain the differences in results. The use of one ME per complexity level (Heyduk, 1975; Burke and Gridley, 1990) makes valid attribution of effects impossible, and synthetic MEs that are unenjoyable (Vitz, 1966; Steck and Machotka, 1975) and dissimilar to real music (Koelsch and Mulder, 2002) provide poor ecological validity. As previously discussed, generative *ad-hoc* accounts of complexity (Heyduk, 1975), and accounts based on formal rules (Steck and Machotka, 1975; Vitz, 1966) have demonstrated low validity or have not been evaluated. The use of subjective ratings of complexity by the same participants who rated familiarity and liking (North and Hargreaves, 1995) disallows attribution of causality among these variables. In conclusion, many previous studies suffer from a potential lack of ecological validity in at least some aspect.

## Conclusions

This study aimed to achieve the highest ecological validity possible for an experimental design, and differed from previous studies by using a naturalistic setting regarding what type of music people would actually listen to, and how they would actually listen to it. This design may come at the expense of losing some generalizability, and further research could expand the range of MEs to an even greater extent.

The lack of an inverse familiarity-complexity relation invalidates the explanation for the secular decrease in music complexity (Serra et al., 2012) proposed in the introduction. Decreasing the level of complexity in newly produced music should not increase turnaround, because even if it attracts more listeners initially, the number of listeners who lose interest should equal those who gain interest, and lead to the same net sales figures. An alternative explanation may be that general intelligence has decreased somewhat in Western populations during this period (e.g., Woodley and Figueredo, 2013; Woodley of Menie et al., 2015a,b; Woodley of Menie and Fernandes, 2015; Madison et al., 2016), and that the industry is catering for the average cognitive processing ability in the largest consumer groups.

While aesthetic theory has typically argued for the existence of optimum levels of complexity and familiarity, the present study found that liking increased monotonically after repeated listening across all levels of complexity. This indicates that familiarity is the single most important predictor for liking of music independent of genre, timbre, structure, complexity and other factors, and that repeated listening can increase the liking of almost any piece of music if listened to under natural circumstances. The results further challenge previous findings of a Wundt curve relationship for liking as a function of repeated listening, because those studies invariably involve less ecological validity and one or more features likely to render very low liking ratings. With the increasing availability of music through new media technologies and at lower costs, exposure and familiarity are likely to play a large and steadily increasing role for listener preferences.

## Ethics statement

This study was carried out in accordance with the recommendations of the Swedish Research Counsil. All participants gave written informed consent in accordance with the Declaration of Helsinki. The protocol was approved by the local Head of studies.

## Author contributions

GS and GM conceived and designed the study, GS conducted the experiments, GM conducted the analyses and wrote the paper.

### Conflict of interest statement

The authors declare that the research was conducted in the absence of any commercial or financial relationships that could be construed as a potential conflict of interest.
