# Supplementary material for: Repeated Listening Increases the Liking for Music Regardless of Its Complexity: Implications for the Appreciation and Aesthetics of Music
Source: Front Neurosci. 2017 Mar 31;11:147. doi: 10.3389/fnins.2017.00147 (PMC5374342; doi:10.3389/fnins.2017.00147)
Supplement: Supplementary file 1 [file DataSheet1.docx]

Appendix A. The 80 music examples subjected to the musicians for the selection study, in rank order of complexity.

| **Rank order** | **Artist** | **Title** | **Style** |
| --- | --- | --- | --- |
| 1 | Bonnie Raitt | Circle dance | Ballad |
| 2 | Air | New Star In The Sky | Ballad |
| 3 | S Dobrogosz & B Andersson | Salieri on the throne | Rock |
| 4 | Genesis | Phret | Pop |
| 5 | Bonnie Raitt | Storm Warning | Rock |
| 6 | Air | Ce Matin La | Progressive rock |
| 7 | Bo Kaspers Orkester | Väljer dig | Pop |
| 8 | J Rudess & Petrucci | Hourglass | Ballad |
| 9 | Air | Kelly Watch the Stars | Rock |
| 10 | Janne Schaffer | Till vinden | Ballad |
| 11 | Bo Kaspers Orkester | Kvarter | Pop |
| 12 | Magnus Edholm Combo | She's Within | Rock |
| 13 | King Crimson | When I say stop, Continue | Progressive rock |
| 14 | Time Design | Two Fifteen | Progressive rock |
| 15 | Genesis | 7 – 8 | Pop |
| 16 | Santana | El Farol | Ballad |
| 17 | Alicia Keys | Rock Wit U | Ballad |
| 18 | L Coryell, S Smith & T Coster | Finale: Wes and Jimi | Progressive rock |
| 19 | Simple Minds | Travelling man | Pop |
| 20 | Bill Bergman | From Now On | Ballad |
| 21 | Up the Creek | Hot Blooded Mama | Rock |
| 22 | The Underground Railroad | The Doorman | Ballad |
| 23 | King Crimson | One Time | Progressive rock |
| 24 | Jonas Knutsson Band | Lemet-Lemet Ánná-Kirste | Jazz |
| 25 | David Sanborn | The Dream | Ballad |
| 26 | Janne Schaffer | Rebeckas Dröm | Ballad |
| 27 | J Rudess & J Petrucci | Track 5 | Ballad |
| 28 | J Rudess & J Petrucci | Bite of the Mosquito | Ballad |
| 29 | David Sylvain & Robert Flipp | Darshan (Road to Graceland) | Progressive rock |
| 30 | David Sanborn | Chicago Song | Ballad |
| 31 | Dream Theater | Pull Me Under | Hardrock |
| 32 | Jonas Knutsson Band | Hymn | World music |
| 33 | Altiplano | Galieros | World music |
| 34 | Vanden Plas | Track 3 | Progressive rock |
| 35 | Nils Landgren Funk Unit | Rock it | Funk |
| 36 | Janne Schaffer | Sång för John och Jimi | Ballad |
| 37 | Nils Landgren Funk Unit | Calvados | Funk |
| 38 | Kenny G | Sade | Ballad |
| 39 | Janne Schaffer | Proggdance | Progressive rock |
| 40 | Jean-Luc Ponty | Happy Robots | Rock |
| 41 | Bill Bergman | The Night begins | Ballad |
| 42 | Peter Seiler | Journey to Nowhere | Progressive rock |
| 43 | Bill Bergman | Midnight Sax Theme | Ballad |
| 44 | Roine Stolt | The Flower King | Progressive rock |
| 45 | The Mike Gibbs Orchestra | Kosasa | World music |
| 46 | Trio con X | Petit Ville | Ballad |
| 47 | Altiplano | Llanganatis | World music |
| 48 | Zero Hour | Stratagem | Progressive rock |
| 49 | Béla Fleck & The Flecktones | The Sinister Minister | Jazz |
| 50 | Trio con X | Pass it On | Jazz |
| 51 | Nils Landgren Funk Unit | Mo stuff | Funk |
| 52 | Dream Theater | Scarred | Hardrock |
| 53 | Trio con X | Chakas dans | Jazz |
| 54 | Dream Theater | Erotomania | Progressive rock |
| 55 | Masque | malaria man | Progressive rock |
| 56 | Altiplano | Bossa de los Andes | World music |
| 57 | Béla Fleck & The Flecktones | New South Africa | Jazz |
| 58 | Greger Wikberg Trio | Svedbergs Massage | Jazz |
| 59 | L Coryell, S Smith & T Coster | First things first | Progressive rock |
| 60 | Trio con X | Killing time | Progressive rock |
| 61 | Béla Fleck & The Flecktones | Stomping Grounds | Progressive rock |
| 62 | Dave Weckl | In Common | Ballad |
| 63 | Dave Weckl | Festival de ritmo | Jazz |
| 64 | Dave Weckl | Here and There | Jazz |
| 65 | Roine Stolt | The Magic Circus of Zeb | Progressive rock |
| 66 | Transatlantic | All of the Above: | Progressive rock |
| 67 | Somnambulist | Troy Built Helen | Progressive rock |
| 68 | Dream Theater | 06:00 | Progressive rock |
| 69 | Dave Weckl | Tower of Inspiration | Jazz |
| 70 | Roine Stolt | The Sounds of Violence | Rock |
| 71 | Itchy Fingers | Teranga | Jazz |
| 72 | L Coryell, S Smith & T Coster | These are odd times | Progressive rock |
| 73 | Janne Schaffer | Bromma Express | Progressive rock |
| 74 | L Coryell, S Smith & T Coster | Bubba | Progressive rock |
| 75 | Béla Fleck & The Flecktones | Vix 9 | World music |
| 76 | Itchy Fingers | £7.50 | Jazz |
| 77 | Itchy Fingers | £7.50 (only saxophone part) | Jazz |
| 78 | Dave Weckl | Softly, As in a Morning Sunrise | Pop |
| 79 | Béla Fleck & The Flecktones | Blu-Bop | World music |
| 80 | Janne Schaffer | Hot Days and Summer Nights | Progressive rock |

Appendix B. The 40 music examples used as stimuli in the listening experiment, sorted according to level of complexity.

|  | **Artist** | **Titel** | **Genre** |
| --- | --- | --- | --- |
|  |  |  |  |
|  | **Complexity level 1** | |  |
| 1 | Bonnie Raitt | Circle dance | Ballad |
| 2 | Air | New Star In The Sky | Ballad |
| 3 | Steve Dobrogosz & Berit Andersson | Salieri on the throne | Rock |
| 4 | Genesis | Phret | Pop |
| 5 | Air | Ce Matin La | Progressive rock |
| 6 | Bo Kaspers Orkester | Väljer dig | Pop |
| 7 | Jordan Rudess & John Petrucci | Hourglass | Ballad |
| 8 | Air | Kelly Watch the Stars | Rock |
| 9 | Bo Kaspers Orkester | Kvarter | Pop |
| 10 | Magnus Edholm Combo | She's Within | Rock |
|  |  |  |  |
|  | **Complexity level 2** | |  |
| 11 | Larry Coryell, Steve Smith & Tom Coster | Finale: Wes and Jimi | Progressive rock |
| 12 | Simple Minds | Travelling man | Pop |
| 13 | Bill Bergman | From Now On | Ballad |
| 14 | Up the Creek | Hot Blooded Mama | Rock |
| 15 | Jonas Knutsson Band | Lemet-Lemet Ánná-Kirste | World music |
| 16 | David Sanborn | The Dream | Ballad |
| 17 | Jordan Rudess & John Petrucci | Bite of the Mosquito | Ballad |
| 18 | David Sylvain & Robert Flipp | Darshan (Road to Graceland) | Progressive rock |
| 19 | David Sanborn | Chicago Song | Ballad |
| 20 | Dream Theater | Pull Me Under | Hardrock |
|  |  |  |  |
|  | **Complexity level 3** | |  |
| 21 | Jean-Luc Ponty | Happy Robots | Rock |
| 22 | Bill Bergman | The Night begins | Ballad |
| 23 | Peter Seiler | Journey to Nowhere | Progressive rock |
| 24 | Bill Bergman | Midnight Sax Theme | Ballad |
| 25 | Roine Stolt | The Flower King | Rock |
| 26 | Béla Fleck & The Flecktones | The Sinister Minister | Jazz |
| 27 | Trio con X | Chakas dans | Progressive rock |
| 28 | Béla Fleck & The Flecktones | New South Africa | World music |
| 29 | Greger Wikberg Trio | Svedbergs Massage | Jazz |
| 30 | Larry Coryell, Steve Smith & Tom Coster | First things first | Progressive rock |
|  |  |  |  |
|  | **Complexity level 4** | |  |
| 31 | Dave Weckl | Tower of Inspiration | Jazz |
| 32 | Itchy Fingers | Teranga | Jazz |
| 33 | Janne Schaffer | Bromma Express | Progressive rock |
| 34 | Larry Coryell, Steve Smith & Tom Coster | Bubba | Progressive rock |
| 35 | Béla Fleck & The Flecktones | Vix 9 | Progressive rock |
| 36 | Itchy Fingers | £7.50 | Jazz |
| 37 | Itchy Fingers | £7.50 (only saxophone part) | Jazz |
| 38 | Dave Weckl | Softly, As in a Morning Sunrise | Jazz |
| 39 | Béla Fleck & The Flecktones | Blu-Bop | Jazz |
| 40 | Janne Schaffer | Hot Days and Summer Nights | Progressive rock |
